# Breast lesions excised via vacuum-assisted system: could we get any clues for B3 lesions before excision biopsy?

**DOI:** 10.1186/s12885-021-08382-7

**Published:** 2021-05-29

**Authors:** Liang Zheng, Fufu Zheng, Zhaomin Xing, Yunjian Zhang, Yongxin Li, Hongbiao Xu, Yuanhui Lai, Jie Li, Wenjian Wang

**Affiliations:** 1 Laboratory of Department of Surgery, the First Affiliated Hospital of Sun Yat-sen University, Guangzhou, 510080 Guangdong China; 2 Department of Thyroid and Breast Surgery, the First Affiliated Hospital of Sun Yat-sen University, Guangzhou, 510080 Guangdong China; 3 Department of Urology, the First Affiliated Hospital of Sun Yat-sen University, Guangzhou, 510080 Guangdong China; 4 Department of Vascular Surgery, the Affiliated Hospital of Qingdao University, Qingdao, 266000 Shandong China

**Keywords:** B3 breast lesion, Uncertain malignant potential, Vacuum-assisted excision biopsy, Breast ultrasonography, Malignancy underestimation

## Abstract

**Background:**

The purpose of this study was to determine the validity of the ultrasound features as well as patient characteristics assigned to B3 (uncertain malignant potential) breast lesions before vacuum-assisted excision biopsy (VAEB).

**Methods:**

This study population consisted of 2245 women with breast-nodular abnormalities, which were conducted ultrasound-guided VAEB (US-VAEB). Patient’s clinical and anamnestic data and lesion-related ultrasonic feature variables of B3 captured before US-VAEB were compared with those of benign or malignant cases, using histopathological results as a benchmark.

**Results:**

The proportions of benign, B3 and malignant breast lesions diagnosed post-US-VAEB were 88.5, 8.2 and 3.4% respectively. B3 high frequent occurred in BI-RADS-US grade 3 (7.7%), grade 4a (11.0%) and grade 4b (9.1%). The overall malignancy underestimation rate of B3 was 4.4% (8/183). Malignant lesions were found mostly in the range of BI-RADS grade 4b (27.3%), grade 4c (33.3%) and grade 5 (100%). Multivariate binary logistic regression analyses (B3 vs benign) showed that non-menopausal patients (95% CI 1.628–8.616, *P* = 0.002), single (95% CI 1.370–2.650, *P* = 0.000) or vascularity (95% CI 1.745–4.150, *P* = 0.000) nodules in ultrasonic features were significant risk factors for B3 occurrences. In addition, patients elder than 50 years (95% CI 3.178–19.816, *P* = 0.000), unclear margin (95% CI 3.571–14.119, *P* = 0.000) or suspicious calcification (95% CI 4.010–30.733, *P* = 0.000) lesions were significantly associated with higher risks of malignant potentials for B3 cases (malignant vs B3).

**Conclusion:**

The results of this study indicate that ultrasound findings and patients’ characteristics might provide valuable information for distinguishing B3 lesions from benign breast abnormalities before VAEB, and help to reduce malignancy underestimation of B3.

## Highlights

- For the first time, we found that breast lesions distributed in BI-RADS-US grade 3-4b presenting with one or more factors of non-menopausal patients, single or vascularity nodules were significantly associated with risks of B3 occurrences.
- B3 cases diagnosed post-US-VAEB, which were classified in the range of BI-RADS-US grade 4b-5 before VAEB, with one or more factors of patients elder than 50 years, unclear margin or suspicious calcification lesions, were remarkably related to risks of malignant potentials.

## Background

Over the last decades, incidence rates of breast cancers in women have been significantly rising. Moreover, breast cancer is the most frequently diagnosed cancer and is also the leading cause of death from cancer in the world as well as in China [1, 2]. For the reasons, breast changes are the principal cause of anxiety in patients who consult physicians in outpatient [3]. In addition to history and physical examination, diagnostic imaging is often utilized by physicians to evaluate the malignant potentials of the breast abnormalities.

Since the initial release of the BI-RADS lexicon for ultrasound (BI-RADS-US) in 2003 [4], together with the rapid development of technique, ultrasound is getting more used for breast screening, especially in Asian women with dense breasts [5]. Compare to mammography, ultrasound examination is a portable, real-time, non-invasive, non-radiative, inexpensive, and highly reproducible method, and well accepted by patients [5–7]. BI-RADS-US is confirmed to be feasible to offer typical features for benign or malignant breast lesions [8, 9]. Therefore, BI-RADS-US is often recognized as the qualified indication of biopsy or surgical excision for breast lesions [10]. Currently, US-VAEB tends to be regarded as the best diagnostic way to differentiate benign from malignant breast changes seen at imaging and is popular in China [11, 12]. VAEB could replace core needle biopsy and open surgical biopsy for diagnoses of breast diseases and treatment of benign breast lesions [13, 14]. It has several advantages, including the ability to get more samples for a more reliable histological diagnosis, the ability to complete removal of the breast lesion, and performance under the real-time guidance of ultrasound [15]. The biopsy diagnoses can be usually categorized as normal/benign, B3 (uncertain malignant potential), or malignant [16, 17]. BI-RADS-US features of typical benign or malignant breast lesions are well concordance with the histological results of biopsies, which could help physicians/patients make choices easier before excision biopsy. However, B3 lesion is usually regarded as a post-VAEB histological diagnosis because few studies are referring to imaging features specifically assigned to B3 before VAEB. Therefore, the diagnosis of B3 is much more challenging in clinical practice. It is reported that the approximate proportions of each biopsy results are normal/benign 70–98.89%, B3 < 10%, malignant < 2%, respectively [18–20]. The data indicate that most breast changes need not excision biopsy. However, in clinical practice, to some extent, the trend of VAEB is to avoid malignancy underestimation of B3, which is subjective and depends on doctors’ individual experience. With increasing concern about over-diagnosis and over-treatment through breast screening, it is regarded as a time to consider the possibility of B3 identification before VAEB.

This study aims to evaluate and compare the common features of ultrasound findings before US-VAEB as well as patient characteristics of B3 cases to those of normal or malignant cases based on histopathological results from US-VAEB.

## Methods

### Study design and patient population

The Institute Research Ethics Committee of the First Affiliated Hospital of Sun Yat-sen University granted permission for this retrospective study. Written informed consent was obtained from every patient for the use of the medical records for research purposes.

All methods were carried out in accordance with relevant guidelines and regulations.

A total of 2245 cases with breast nodules were performed US-VAEB in our institute, from June 2014 to December 2018. The inclusion criteria were as follows: (1) All patients included in the study group were women with a US-detectable breast lesion; (2) Availability of clinical and anamnestic data of patients, and ultrasound imaging reporting and data system lexicon for the breast lesions before US-VAEB; (3) The first time of US-VAEB; (4) Availability of histopathological report; (5) All cases were imaging followed up for at least 12 months after US-VAEB. No follow-up cases were excluded.

### Variables

Clinical data for statistical analyses included patient ages at treatment (range 12–79 years), reproductive ages, menopause, the complaint of pain, palpable nodules, lesions in the left or right breast, and quadrant of lesions. Menopause usually was defined retrospectively as 12 months of amenorrhoea accompanying with or without menopausal symptoms [21]. Reproductive age comprised 15–49 years without abnormal menstruation [21]. Sonographic characteristics for the lesions were assessed referring to the American College of Radiology (ACR) Breast Imaging Reporting and Data System (BI-RADS) Atlas Fifth Edition [22], which included nodule multifocality, shape, orientation, margin, echo pattern, suspicious calcification, architectural distortion, duct changes, vascularity, skin changes, and lymph nodes. Posterior features were excluded because most cases missed the related information. In addition, orientation, architectural distortion and skin changes were rarely observed in the study cohort. And they did not meet the statistic analysis condition. Here, echo pattern comprised anechoic, hyperechoic, complex cystic and solid, hypoechoic or heterogeneous. Suspicious calcification comprised intra mass calcification and/or intra ductal calcification. Vascularity included internal vascularity and/or vessels in rim.

### Data analysis

The SPSS 25.0 software package was used for all statistical calculations. Chi-square test / Fisher’s exact test was utilized to compare the differences of ultrasonic features of B3 lesions and clinical and anamnestic data of the associated patients with those of benign or malignant cases. Univariate and multivariate binary logistic regression analyses were used to find independent predictive risk factors for B3 or malignant potentials. The result with *P* value < 0.05 was considered statistically significant.

## Results

### Study population description

Our database included 2245 women who had recent-onset US-detectable breast findings. The mean age was 37.5 years (range 12–79 years). The most frequent complaint of patients was palpable nodules (86.8%). The average diameter of the lesions was 13.2 mm (range 2–75 mm). The less frequent complaints were pain (4.1%) and nipple discharge (1.2%), respectively. And 7.9% of cases are asymptomatic, which were image findings. All patients were addressed to undergo US-VAEB for the first time at our institute. T he median duration of imaging follow-up post-US-VAEB was 24.7 months (range 12.0–60.0 months).

The clinicopathologic characteristics of the cases are summarized in Table 1.

**Table 1:** The distributions of benign, B3 or malignant breast lesions according to BI-RADS-US

BI-RADS category	Total	Benign	Rate (%)	B3	Rate (%)	Malignant	Rate (%)
0	5	3	60	2	40	0	0
2	141	138	97.9	3	2.1	0	0
3	1735	1568	90.4	134	7.7	33	1.9
4a	291	235	80.8	32	11.0	24	8.2
4b	44	28	63.6	4	9.1	12	27.3
4c	21	14	66.7	0	0	7	33.3
5	8	0	0	0	0	8	100

*BI-RADS* the Breast Imaging Reporting and Data System, *US* ultrasound

### BI-RADS-US category assessment based on histopathological results of VAEB

Post-US-VAEB histopathological diagnoses confirmed that there were benign, B3, and malignant breast lesions as 1986 (88.5%), 183 (8.2%), and 76 (3.4%), respectively. Malignant lesions account for the minimum amounts of VAEB cases, and the overwhelming majority of VAEB cases are nonmalignant disorders that are unnecessarily subjected to biopsy, which is consistent with previous reports [23, 24]. Afterward, we subclassified the lesions according to their BI-RADS-US category assessment. The incidences of B3 and malignant breast lesions in each grade of BI-RADS-US were listed in Table 1. Notably, the prevalent range for B3 was in BI-RADS-US grade 3 (7.7%), grade 4a (11.0%), and grade 4b (9.1%). And they were rarely diagnosed in BI-RADS-US grade 4c or grade 5. On the other hand, the malignant lesions were mainly distributed in the range of BI-RADS-US grade 4b (27.3%), grade 4c (33.3%), and grade 5 (100%), which are compatible with the literature [25].

### Malignancy underestimation rate and recurrence rate of B3

Initially, there was a total of 183 cases of B3 lesions proved post-US-VAEB. The histopathological results of B3 subtypes were listed in Table 2. Meanwhile, one case of atypical ductal hyperplasia (ADH) which could not be excluded from the possibility of ductal carcinoma in situ (DCIS) was finally confirmed to be invasive breast cancer by open surgery (OS). One case of complex sclerosing lesions/radial scars (CSL/RS) which was discordant with image finding was confirmed to be invasive breast cancer through OS. One case of papillary lesions/atypical ductal hyperplasia (PL/ADH) which had a higher risk of malignant potential was received OS subsequently and verified to be intraductal carcinoma with lobular carcinoma [26]. Besides, 5 cases were followed up after VAEB, and found recurrences at the sites of operation, which were demonstrated as malignancies via OS, including 2 cases of ADH confirmed to be DCIS and invasive ductal carcinoma respectively, 2 cases of PL confirmed to be DCIS and invasive ductal carcinoma, 1 case of phyllodes tumors (PT) diagnosed as invasive breast cancer. In general, the cumulative risk of malignancy underestimation in the B3 cohort was 4.4% (8/183). The individual malignancy underestimation rates of B3 subtypes were displayed in Table 2. It is noteworthy that the most often underrated B3 subtypes were PL/ADH 50% (1/2), ADH 23.1% (3/13), and CSL/RS 16.7% (1/6). The results are in line with the reports [26, 27]. Although the malignancy underestimation rate of B3 is limited, multidisciplinary communication and imaging follow-up are necessary so as not to miss malignant potential [28].

**Table 2:** Malignancy underestimation rates of B3 subtypes

B3 subtype	Number	Underestimation	Rate (%)
ADH	13	3	23.1
FEA	4	0	0
LN	3	0	0
PL	84	2	2.4
PL/ADH	2	1	50
PT	71	1	1.4
CSL/RS	6	1	16.7

*ADH* atypical ductal hyperplasia, *FEA* flat epithelial atypia, *LN* classical lobular neoplasia, *PL* papillary lesions, *PL*/*ADH* papillary lesions/atypical ductal hyperplasia, *PT* phyllodes tumors, *CSL*/*RS* complex sclerosing lesions/radial scars

After the period of follow-up, the recurrence rates of B3 subtypes were summarized in Table 3. We noticed that the highly frequent recurrences of B3 subtypes were ADH 10% (1/10), PT 8.6% (6/70), and PL 4.9% (4/82). The recurrence rates of the later two subtypes are concordant with the literature [29, 30]. Whether the higher recurrence rate of ADH was due to its fewer cases needs further observation.

**Table 3:** Recurrence rates of B3 subtypes

B3 subtype	Number	Recurrence	Rate (%)
ADH	10	1	10
FEA	4	0	0
LN	3	0	0
PL	82	4	4.9
PL/ADH	1	0	0
PT	70	6	8.6
CSL/RS	5	0	0

*ADH* atypical ductal hyperplasia, *FEA* flat epithelial atypia, *LN* classical lobular neoplasia, *PL* papillary lesions, *PL*/*ADH* papillary lesions/atypical ductal hyperplasia, *PT* phyllodes tumors, *CSL*/*RS* complex sclerosing lesions/radial scars

### The specific ultrasonic features and patients’ characters of B3 comparing to those of benign or malignant cases

In clinical practice, uncertain malignant potentials are the most frequent causes which disturb both patients and physicians. To a certain extent, this could explain why most of the biopsy cases are nonmalignant breast disorders. One question is presented here that could we get any clues for B3 changes before the excision biopsy? Therefore, we analyzed the differences between the ultrasonic features corresponding to B3 lesions before VAEB and their associated patients’ characteristics and those of benign or malignant cases by Chi-square test / Fisher’s exact test. Negative statistic analyses included the complaint of pain, palpable nodules, lesions in left or right, quadrant of lesions, echo pattern, multifocality, duct changes and lymph nodes. To our best knowledge, it’s the first time that we noticed that among B3 cases, the incidences of non-menopausal patients (*P* = 0.002), single (*P* = 0.000) or vascularity nodules (*P* = 0.000) in ultrasound findings were significantly increased, compared to those of benign cases. Also, the incidence of the irregular shape of nodules in B3 cases tended to be higher than that of benign cases (*P* = 0.080) (Table 4). In the meantime, we compared malignant cases with B3 cases and found that the incidences of patients elder than 50 years (*P* = 0.000), non-reproductive age (*P* = 0.000) and menopause (*P* = 0.000), and lesions with irregular shape (*P* = 0.000), uncircumscribed margin (*P* = 0.000), vascularity (*P* = 0.002) or suspicious calcification (*P* = 0.000) in ultrasound findings were significantly increased in malignant cases (Table 4). Furthermore, both univariate and multivariate binary logistic regression analyses (B3 vs benign) showed that non-menopausal patients, single or vascularity nodules were significant risk factors for B3 occurrences (Table 5). While malignant compared to B3, univariate binary logistic regression analyses showed that patients elder than 50 years, and unclear margin, vascularity or suspicious calcification lesions were closely associated with malignancies. Moreover, multivariate logistic regression analyses demonstrated that patients elder than 50 years, unclear margin or suspicious calcification lesions were significant risk factors of malignant potential for B3 cases (Table 6). On these grounds, we reviewed the 8 malignancy underestimation cases in the B3 cohort and found that they presented with at least one or more malignant risk factors.

**Table 4:** Comparison between characteristics of B3 cases and those of benign or malignant cases

	Benign	B3	***χ***^**2**^	***P***	B3	Malignant	***χ***^**2**^	***P***
**Age**								
≤ 50	1809	165	2.081	0.149	165	56	34.583	0.000
> 50	177	10			10	28		
**Pain**								
No	1913	164	2.93	0.087	164	76	0.88	0.35
Yes	73	11			11	8		
**Duct changes**								
No	1912	168	0.033	0.86	168	80	0.081	0.78
Yes	74	7			7	4		
**Lymph nodes**								
No	1950	170	0.943	0.33	170	81	0.097	0.76
Yes	36	5			5	3		
**Palpable**								
Yes	1727	151	0.06	0.8	151	70	0.40	0.53
No	259	24			24	14		
**Left/right**								
Left	998	92	0.35	0.56	92	40	0.56	0.46
Right	988	83			83	44		
**Quadrant**								
Areola	57	10	6.61	0.16	10	2	8.59	0.07
Upper outer	939	74			74	40		
Upper inner	549	45			45	30		
Lower inner	189	21			21	3		
Lower outer	252	25			25	9		
**Echo pattern**								
Anechoic	169	12	2.8	0.60	12	4	2.0	0.74
Hyperechoic	113	8			8	4		
Complex cystic/solid	141	13			13	3		
Hypoechoic	1491	132			132	68		
Heterogeneous	72	10			10	5		
**Reproductive age**								
Yes	1148	97	0.372	0.542	97	25	15.006	0.000
No	838	78			78	59		
**Menopause**								
No	1767	169	9.955	0.002	169	58	39.706	0.000
Yes	219	6			6	26		
**Multifocality**								
Unifocal	454	65	17.979	0.000	65	35	0.490	0.555
Multifocal	1532	110			110	49		
**Shape**								
Regular	1786	150	3.064	0.080	150	48	25.733	0.000
Irregular	200	25			25	36		
**Margin**								
Circumscribed	1611	145	0.319	0.572	145	29	60.137	0.000
Not circumscribed	375	30			30	55		
**Suspicious calcification**								
Absent	1864	168	1.316	0.572	168	56	41.785	0.000
Present	122	7			7	28		
**Vascularity**								
Absent/scarce	1847	142	30.870	0.000	142	53	9.937	0.002
Present	139	33			33	31

**Table 5:** Binary logistic regression analyses between characteristics of benign cases and those of B3 cases

	Univariate		Multivariate	
	OR (95% CI)	***P***	OR (95% CI)	***P***
**Menopause**				
Yes	3.491 (1.528, 7.976)	0.003	3.745 (1.628, 8.616)	0.002
No				
**Multifocality**				
Multifocal	1.994 (1.442, 2.757)	0.000	1.905 (1.370, 2.650)	0.000
Unifocal				
**Shape**				
Regular	1.488 (0.951, 2.329)	0.082	1.259 (0.788, 2.014)	0.336
Irregular				
**Vascularity**				
Absent/scarce	3.088 (2.037, 4.682)	0.000	2.691 (1.745, 4.150)	0.000
Present				

*95% CI* 95% confidence interval, *OR* odds ratio

**Table 6:** Binary logistic regression analyses between characteristics of malignant cases and those of B3 cases

	Univariate			Multivariate	
	OR (95% CI)		***P***	OR (95% CI)	***P***
**Age**					
≤ 50	8.25 (3.770, 18.053)		0.000	7.936 (3.178, 19.816)	0.000
> 50					
**Margin**					
Circumscribed	9.167(5.044, 16.659)		0.000	7.101 (3.571, 14.119)	0.000
Not circumscribed					
**Microcalcification**					
Absent	12.000 (4.969, 28.981)		0.000	11.101 (4.010, 30.733)	0.000
Present					
**Vascularity**					
Absent/scarce	2.517(1.405, 4.509)		0.001	1.711 (0.808, 3.623)	0.161
Present					

*95% CI* 95% confidence interval, *OR* odds ratio

## Discussion

B3 lesions are borderline with either benign or malignant breast disorders. Women with these lesions are often suffering from psychological depression because of an increased risk of finding concomitant cancer, or evolution toward in situ or invasive cancer over a long time [26, 30]. The patients that underwent a breast biopsy claimed that they also have experienced biopsy-related stress even if the results were nonmalignant [31].

While concern about over-diagnosis and over-treatment through breast screening is on the increase, it must be considered the strategies to manage breast abnormalities because the approaches differences are not trivial and could translate into possibly thousands of patients undergoing unnecessary biopsies or an equal number with delays in diagnoses of malignancies [28].

Our data showed that the overwhelming majority of histopathological results of the 2245 US-VAEB cases were benign breast disorders (88.5%), and only a small proportion (3.4%) was malignancies. The results are consistent with the literature [11, 18–20]. It shows that criteria should be more strict to avoid unnecessary VAEB procedures for the patient’s clinical and economic benefit unless there is a specific request from the patient. Our data showed that 8.2% of the 2245 VAEB cases were proved to be B3 lesions. The overall malignancy underestimation rate for this clinical dilemma was 4.4%. The data suggested that excision biopsies are not needed for most of these borderline changes as well. Furthermore, we noticed that B3 high frequently occurred in the range of BI-RADS-US 3-4b, which is partly consistent with the reports [31, 32]. And the data showed that the most often underrated B3 subtypes were PL/ADH (50%), ADH (23.1%), and CSL/RS (16.7%), respectively. Moreover, the most frequent recurrence rates of B3 subtypes were ADH (10%), PT (8.6%), and PL (4.9%). These are compatible with the previous study [33]. In a word, taking into account both the incidence and the overall malignancy underestimation rate of B3 lesions, only a minority of B3 cases should be subjected to VAEB. If consideration on B3 subtypes’ malignancy underestimation rates and their recurrence rates, we recommend re-biopsy/OS for PL/ADH, ADH, and PT subtypes, which is in accordance with the reports [29, 34, 35].

At present, several studies refer to ultrasonic features before VAEB corresponding to B3 lesions and their associated patients’ characteristics but they all are either lack statistical analyses or categorizing B3 into normal lesions for analyses [15, 23, 36]. Therefore, the data could hardly help to identify B3 from benign or malignant breast changes. For the first time, we found that the incidences of non-menopausal patients, single or vascularity nodules were significantly increased in B3 cases compared to those of benign cases. Besides these, the incidence of nodules with irregular shape in B3 cases tended to be higher than that of normal cases (*P* = 0.080). Then, multivariate logistic regression analyses demonstrated that non-menopausal women, single or vascularity nodules were significant risk factors for B3 cases. Combining with the above mentioned, it indicated that breast lesions in the range of BI-RADS-US grade 3-4b presenting with one or more of the above risk factors before VAEB should be aware of B3 occurrences. On the other hand, compared to B3 cases, the incidences of patients elder than 50 years, non-reproductive age and menopause, and irregular shape, uncircumscribed margin, vascularity or suspicious calcification lesions were significantly increased in malignant cases. Furthermore, multivariate logistic regression analyses showed that patients elder than 50 years, unclear margin or suspicious calcification lesions were significant risk factors of malignant potential for B3 cases. It suggested that the B3 cases confirmed post-US-VAEB, which were classified into BI-RADS-US grade 4b-5 as we discussed earlier, with one or more of the above malignant risk factors should be watched out for malignancy underestimation.

### Study limitations

The first is the fewer cases of the B3 cohort and the retrospective nature of the study. The second limitation is that there is a lack of ultrasonic features of B3 subtypes and their associated patients’ characteristics which allow more accurate comparisons between B3 and benign or malignant cases. Finally, this study did not analyze the influence of the family history of breast cancer on first-degree relatives, and the influence of age as a continuous variable in point of malignancy risk factors [37, 38]. Hence, further studies are required in these regards.

## Conclusions

The vast majority of cases conducted US-VAEB were proved to be benign breast changes. The cases assigned in the range of BI-RADS-US grade 3-4b with one or more factors of non-menopausal patients, single or vascularity nodules should be cautious of B3 occurrences. For these cases, close clinical and imaging follow-up is recommended. The B3 cases confirmed post-US-VAEB, which were classified into BI-RADS-US grade 4b-5, with one or more factors of elder than 50 years, unclear margin or suspicious calcification lesions were remarkably related to malignant potential. For these cases, interval re-biopsy/OS should be considered subsequently post-US-VAEB to avoid malignancy underestimation. If confirmed on larger series and prospectively validated, the results could help to improve strategies to identify B3 from benign breast abnormalities before VAEB and reduce the malignancy underestimation of B3.

## Data Availability

All data generated or analyzed during this study are included in this published article.

## Abbreviations

B3: Uncertain malignant potential
VAEB: Vacuum-assisted excision biopsy
US: Ultrasound
DCIS: Ductal carcinoma in situ
ADH: Atypical ductal hyperplasia
FEA: Flat epithelial atypia
LN: Classical lobular neoplasia
PL: Papillary lesions
PL/ADH: Papillary lesions/atypical ductal hyperplasia
PT: Phyllodes tumors
CSL/RS: Complex sclerosing lesions/radial scars
95% CI: 95% confidence interval
OR: Odds ratio
